# Histological findings of corneal tissue after failed phototherapeutic keratectomy in macular corneal dystrophy – a case report

**DOI:** 10.1186/s12886-022-02400-3

**Published:** 2022-05-08

**Authors:** Caroline Julia Gassel, Jens Martin Rohrbach, Daniel Röck, Karl Ulrich Bartz-Schmidt, Tobias Röck

**Affiliations:** 1 Centre for Ophthalmology, University Eye Hospital, Eberhard Karls University Tübingen, Elfriede-Aulhorn-Str. 7, 72076 Tübingen , Germany; 2 Prof. Dr. med. Tobias Röck, Centre for Ophthalmology, University Eye Hospital, Eberhard Karls University Tübingen, Elfriede-Aulhorn-Str. 7, 72076 Tübingen, Germany

**Keywords:** Macular corneal dystrophy, Phototherapeutic keratectomy, Penetrating keratoplasty, Case report, Corneal dystrophy

## Abstract

**Background:**

Macular corneal dystrophy is a rare inherited disease of the cornea leading to deposits mainly in the stroma. Affected patients suffer from progressive loss of visual acuity which should be treated with penetrating keratoplasty.

This is the first case report describing the clinical and histopathological findings of corneal tissue after failed phototherapeutic keratectomy (PTK) in a patient with macular corneal dystrophy.

**Case presentation:**

A 32-year-old man presented with visual impairment, blurred vision and increasing glare sensitivity in both eyes in 2014. All symptoms had existed for several years and had recently increased sharply. A corneal dystrophy was diagnosed and penetrating keratoplasty was recommended but the patient was hesitant to undergo surgery. In 2018, in contrast to current guidelines, a PTK was performed in both eyes in Turkey for unknown reasons. In May 2019, he presented again in our clinic. Best corrected visual acuity was markedly reduced in both eyes. Slit-lamp examination revealed multiple dense, poorly circumscribed grey-white patchy changes in the stroma accompanied by corneal opacity in both eyes. In February 2020, the patient decided to have penetrating keratoplasty performed at the University Eye Hospital in Tübingen. The explanted cornea was stained for acid mucopolysaccharides (AMP) and periodic acid–Schiff staining (PAS). The histopathological examination revealed destruction of Bowman’s layer and a subepithelial fibrosis band due to the PTK previously performed. The AMP staining demonstrated blue deposits typical of macular corneal dystrophy, mainly in the stroma but also in the endothelium. Interestingly, the acidic mucopolysaccharides were found increased in the PTK-induced subepithelial fibrosis band. The postoperative course after keratoplasty was favourable with a significant increase in visual acuity and a clear graft.

**Conclusions:**

This report presents the first case of a histologically evident exacerbation of macular corneal dystrophy after PTK and emphasizes the relevance of thorough pre-interventional diagnosis and patient selection to consider other therapeutic approaches, such as penetrating keratoplasty.

## Background

Corneal dystrophies are rare, inherited disorders of the cornea which can affect different layers of the cornea. They typically occur bilaterally but often asymmetrically and progress slowly. Abnormal material is found deposited in the cornea. Corneal dystrophies are classified as epithelial and subepithelial, epithelial-stromal TGFBI dystrophies, stromal or endothelial dystrophies depending on the anatomic origin and location of the lesions or deposits [1]. Hereditary dystrophies must be distinguished from corneal degenerations that occur secondarily as a result of other factors [2].

Macular corneal dystrophy is categorized as a stromal dystrophy. It is an autosomal recessive disorder of the corneal stroma with an impaired proteoglycan synthesis, often due to a mutation in the *CHST6* gene [3, 4]. Prevalence is higher in Iceland, India and Saudi Arabia [5–7] due to a higher incidence of gene mutations that can trigger MCD and are related to consanguinity [5, 8, 9]. In a US study, a prevalence of 9.7 per 1 million was estimated [10].

In this disorder, abnormal glycosaminoglycans accumulate mainly in the stroma, but also in Bowman’s membrane, Descemet’s membrane, and the endothelium. These deposits affect refractive power and transparency of the cornea. In the long term, this usually leads to bilateral opacities and thinning of the cornea with increasing loss of visual acuity. The stromal opacities spread in the centre and periphery [11].

Histopathologically, intracellular accumulations of glycosaminoglycans are found in keratocytes and endothelial cells. Additionally, extracellular depositions are present in the stroma and Descemet membrane. The Descemet membrane may display guttae [11]. The deposits stain histologically like glycosaminoglycans, for example with periodic acid-Schiff or alcian blue. They also have an affinity for colloidal iron [12]. Electron microscopy reveals fibrillogranular material in small vacuoles in the stroma. In ultrasound biomicroscopy, focal protrusions and loss of continuity of the posterior corneal layer might be detected [13].

Therapeutical management consists of penetrating keratoplasty. Postoperative vision prognosis is good, but recurrences can occur many years after the transplantation. Descemet membrane endothelial keratoplasty is not expedient in macular corneal dystrophy because it will not remove all the pathologically altered tissue. Photo phototherapeutic keratectomy (PTK) has shown to increase visual acuity moderately for a limited period of time in some studies [14].

To our knowledge, this is the first publication describing an exacerbation of macular corneal dystrophy after PTK, leading to severe vision deterioration.

## Case presentation

A 32-year-old man of Turkish origin presented at University Eye Hospital Tübingen (Tübingen, Germany) with visual impairment, blurred vision and increasing photophobia in both eyes. The man reported still good visual acuity in youth and at the time of his driving test. The described symptoms had existed for several years before his presentation and had recently increased sharply. There was no known family history of ophthalmologic disease.

**Fig. 1:**
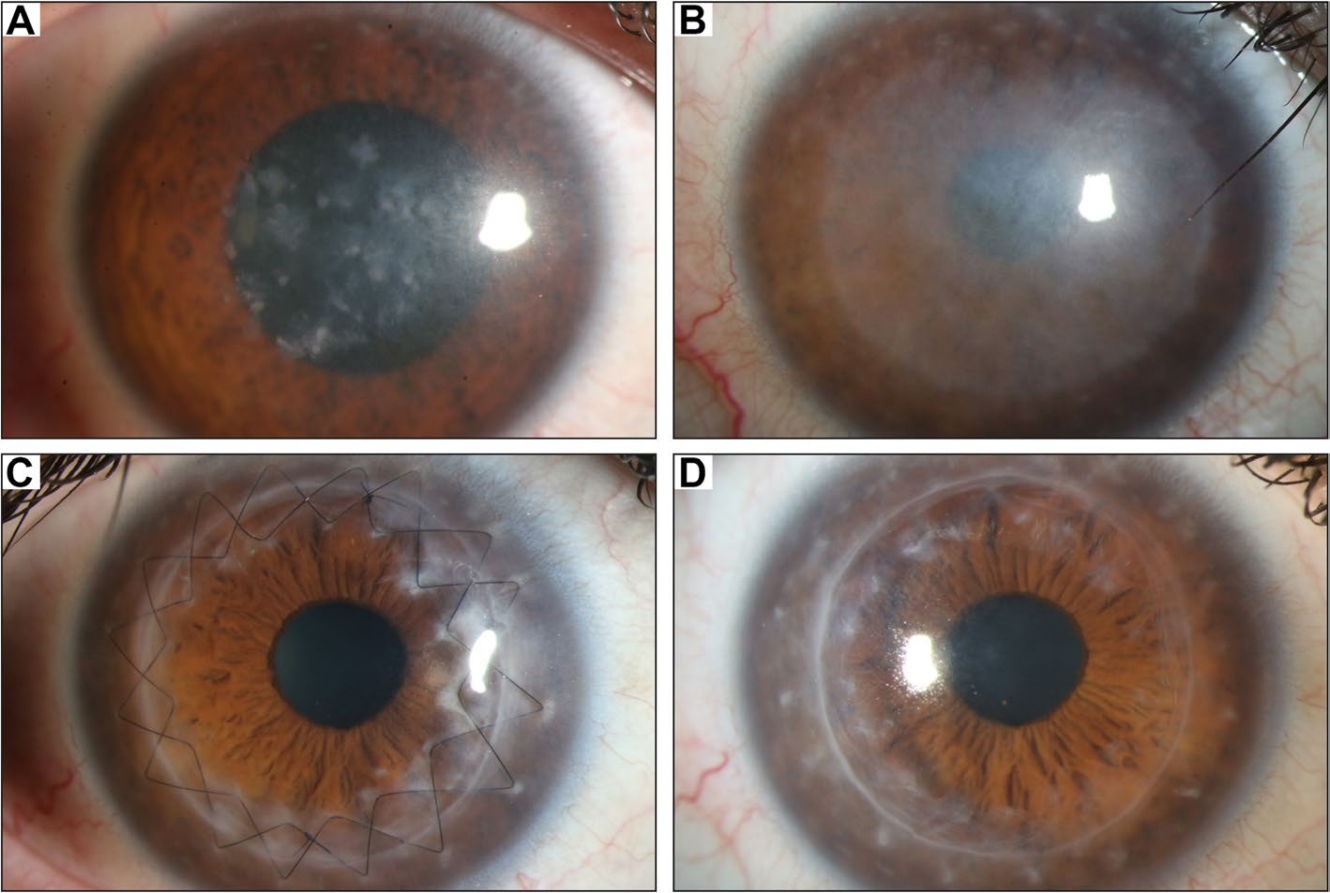
(**A**) Corneal findings of the patient’s OD at first presentation in our clinic in 2014. Subepithelial and stromal depositions and opacities are typical in macular corneal dystrophy. BCVA was 20/50 in the OD and 20/40 in the OS. (**B**) Status of the cornea of the OD in February 2020 before penetrating keratoplasty. In 2019, the patient had a PTK performed in Turkey. Lesions appear to have increased in the area of the PTK and corneal opacity was present. The patient reported a severe vision loss and blurry vision as well as a burning sensation of the eyes. BCVA was decreased to 20/400 in the OD and 20/320 in the OS. (**C**) Cornea of the OD in June 2020, 4 months after penetrating keratoplasty. The graft was clear, fine neovascularisations were visible on the upper corneal rim. BCVA had restored to 20/40 in the OD. In the OS, BCVA was 20/80. (**D**) Cornea of the OD in September 2021, 19 months after penetrating keratoplasty. The two sutures had been removed after 15 and 16 months. BCVA was 20/25 in OD and 20/200 in OS

At first presentation in 2014, best corrected visual acuity (BCVA) was 20/50 in the right eye (OD) and 20/40 in the left eye (OS). The intraocular pressure was in the normotensive range. Slit-lamp (Haag-Streit, Köniz, Switzerland) examination revealed multiple dense, poorly circumscribed grey-white patchy changes in the stroma accompanied by corneal opacity (Fig. 1 A). These findings were consistent with a macular corneal dystrophy. The clinical diagnosis was made based on standard ophthalmological examinations such as the best corrected visual acuity test and slit lamp examination. Photographs of the cornea were taken. We recommended penetrating keratoplasty to improve vision and explained the possible risks. The patient was initially still reluctant to have an operation. Five years later, in May 2019, he presented again and reported a severe loss of vision and blurry vision in both eyes as well as an occasional burning sensation of the eyes. He reported that he had undergone a PTK on both eyes in Turkey in the meantime in 2018. BCVA was 20/400 in the OD and 20/320 in the OS. Corneal opacities were present in both eyes and the lesions were increasingly visible in the area of PTK in both eyes (Fig. 1B).

The patient was placed on the cornea donation waiting list again. In February 2020, the penetrating keratoplasty was performed at our clinic on the patient’s OD under intubation anaesthesia in our clinic. During surgery, a sample of the cornea was taken and prepared for histopathologic examination. Cuts of the corneal specimen were stained with PAS and a staining for AMP. To rule out granular and lattice corneal dystrophies, Masson staining as well as Congo Red staining were performed.

For the AMP staining, the corneal tissue was deparaffinised, treated with 12% acetic acid for 3 min, then placed in Müller’s working solution for 60 min. The specimen was rinsed twice with 12% acetic acid, then left for 20 min in 12% acetic acid, and then left for 20 min in 5% hydrochloric acid + 5% potassium ferrocyanide in a 1:1 ratio. Then it was rinsed with distilled water, treated with 0.1% nuclear red for 5–7 min. This was followed by an ascending alcohol series and then treatment with xylene. The preparation was mounted with Eukitt mounting medium (O-Kindler GmbH, Freiburg, Germany). The PAS, Masson and Congo Red staining were performed in the usual manner [15–17].

**Fig. 2:**
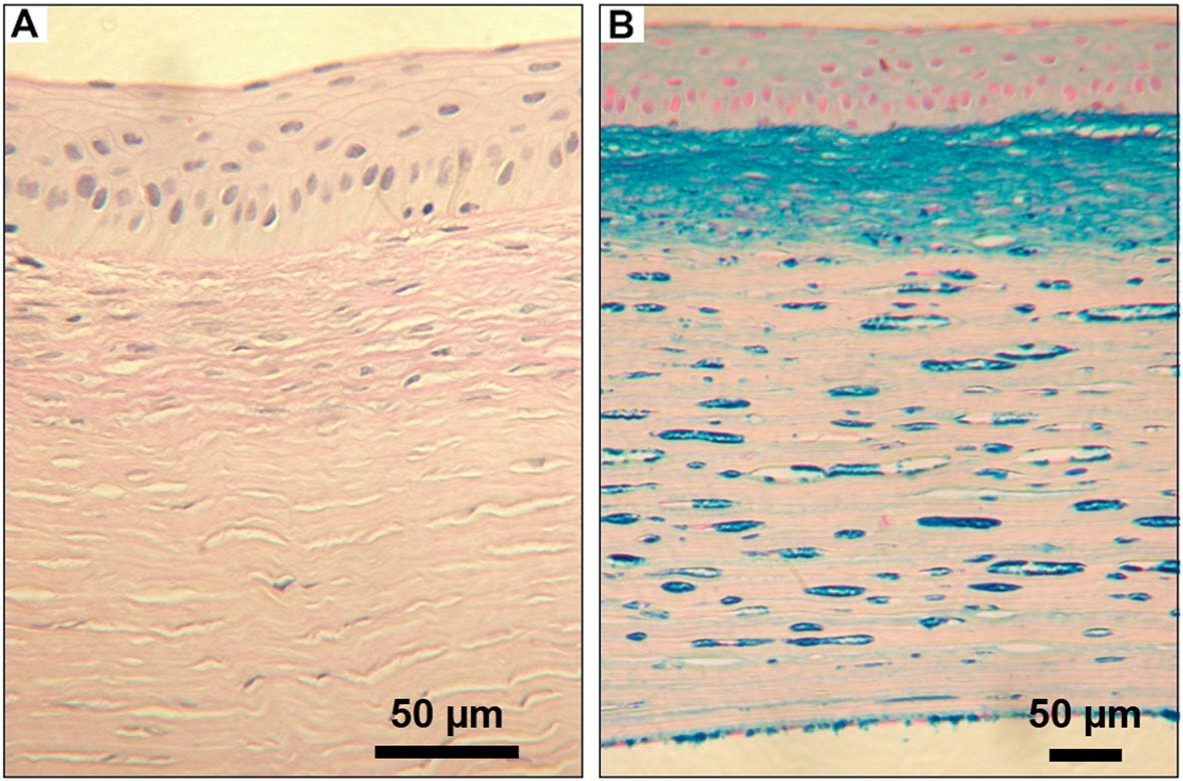
(**A**) Anterior region of the explanted cornea in the centre, PAS staining. The epithelium (upper edge of the image) is largely normally stratified, the Bowman layer is completely destroyed. Subepithelially there is a keratocyte-rich “fibrosis band” as a result of the PTK performed. Unobtrusive stroma at the lower edge of the image. Descemet’s membrane and endothelium are not depicted, but they appear normal. Magnification: x400 (**B**) In AMP staining for acid mucopolysaccharides, the blue deposits typical of macular corneal dystrophy are found mainly in the stroma, but also in the endothelium. Interestingly, the acidic mucopolysaccharides are found increased in the PTK induced “subepithelial fibrosis band”. Masson stain (for granular corneal dystrophy) and Congo red stain (for lattice corneal dystrophy) were negative. Magnification: x200

The histopathologic examination of the corneal specimen revealed a destruction of the Bowman layer and a subepithelial fibrosis band (Fig. 2 A). Descemet membrane and epithelium appeared normal. The AMP staining showed the characteristic blue deposits in the corneal stroma and endothelium (Fig. 2B). The AMPs were much more abundant in the fibrosis band which was probably induced by the PTK. Negative staining with Masson (for granular corneal dystrophy) and Congo red (for lattice corneal dystrophy) helped to rule out these other two entities.

On the first postoperative day, BCVA had increased to 20/60 on the OD. The corneal graft was clear, and the sutures were tight (Fig. 1 C). A bandage contact lens was inserted and left in place for one week. Four months after surgery, BCVA was 20/40 and fine neovascularisations were visible on the upper corneal rim, but not on the graft. Local therapy with ciclosporin A was initiated. 15 months after penetrating keratoplasty, the corneal graft was still clear (Fig. 1D), and the first suture was removed. BCVA was still 20/40 on the OD. The second suture was removed 6 weeks later. The patient’s last presentation in our clinic was in September 2021, 19 months after surgery. The corneal graft was still very clear and BCVA was measured 20/25, which was also reflected in the subjective perception of the patient. The patient’s prognosis is good, and the corneal graft can remain clear for many years. Nevertheless, recurrences of opacities on the graft are possible after several years.

All procedures were in accordance with the Declaration of Helsinki. Informed consent for publication was obtained from the patient.

## Discussion and conclusions

It is unclear why PTK was performed in Turkey. This could not be ascertained from anamnesis either. Possibly, a misdiagnosis could have let to the conduction of the PTK. Possible differential diagnoses where PTK is indicated are epithelial corneal dystrophies.

In this case, we observed a significant clinical and morphological deterioration of the disease after PTK. This was associated with an enhancement of macular corneal dystrophy lesions as proved by histopathological examinations.

In contrast, a retrospective study from 2005 reported increases of BCVA after PTK treatment in superficial corneal opacities caused by macular dystrophy. Nevertheless, the authors addressed that primary penetrating keratoplasty was still the definite therapy for this disorder [14].

In a study from 2006, histologic changes in corneas with macular dystrophy treated by PTK were analyzed. According to the results, the PTK treated corneas were thinner and appeared more regular than the control corneas. Also here, the authors report an accumulation of AMP-positive deposits in the PTK-treated area. After hyaluronic acid digestion, this subepithelial layer of AMP-positive substances disappeared so that the authors assume that the alterations were signs of a haze formation and not a recurrence of the macular dystrophy lesions. Transmission electron microscopy revealed more keratocytes with amorphous inclusions in the anterior stroma compared with the controls [18]. These results are relativised by the fact that in this study only a very small number of cases of 2 eyes was examined. The authors’ conclusion contrasts with our assumption that PTK can enhance the dystrophic changes as observed in the case presented here.

In 2004, Seitz et al. suggest that due to the size and distribution of histopathologic deposits, PTK might be effective to improve visual acuity particularly in patients with granular dystrophy where deposits are located more superficial. For deeper opacities and associated thinning of the cornea, PTK does not appear as beneficial [19, 20]. Since granular corneal dystrophy belongs to the epithelial-stromal TGFBI dystrophies and deposits originate from the epithelium, it is consistent that PTK can be effective in removing the deposits especially in early stages of granular dystrophy. In more advanced stages of granular dystrophy, lesions can be distributed over all corneal layers and PTK treatment will not be sufficient.

In macular corneal dystrophy, deposits derive from and are located in the stroma. Thus, it is explainable that PTK might not be effective here.

Consistent with this, good long-term results after excimer laser PTK were observed in patients with Salzmann’s nodular degeneration, where the nodules are subepithelial or in the Bowman’s layer [21]. In 2016, a good BCVA recovery after pannus removal combined with excimer laser PTK was observed in a retrospective study. The authors attributed this in part to a myopic shift induced by PTK in those patients [22]. Successful treatments of recurrent erosions by excimer laser PTK have also been described in Map-Dot fingerprint dystrophy which is classified as an epithelial basement membrane dystrophy [23].

Interestingly, in a genotype phenotype study from 2008 where a family of macular dystrophy patients was genetically tested and different mutations of the *CHST6* gene were detected, the authors suggest that therapeutic management should even depend on the particular genetic mutation. They observed that especially frameshift mutations of *CHST6* cause more affected phenotypes of macular dystrophy with deeper deposits [24]. In these cases, penetrating keratoplasty should primarily be preferred to PTK since PTK is limited to superficial corneal lesions. It must be noted that recurrences of macular corneal dystrophy are to be expected after a period of several years after penetrating keratoplasty. In patients with more superficial location of the macular deposits, PTK might be an effective primary treatment option to restore visual acuity, especially as prior PTK does not seem to influence the outcome of subsequent penetrating keratoplasty [25].

In conclusion, PTK can be an effective therapy in corneal dystrophies where the deposits originate from the epithelium and lesions are located superficially. However, the case presented in this report shows that macular corneal dystrophy with deeper stromal deposits should not be treated by PTK and can even lead to enhancement of the pathogenic lesions. This observation was proved by histopathologic examination of corneal tissue. Therefore, the correct diagnosis of the corneal dystrophy is crucial to guide the patient to the right therapy. In cases of macular corneal dystrophy, penetrating keratoplasty represents the gold standard and can lead to a significant BCVA improvement, even if a recurrence of the lesions is possible after years.

## Data Availability

The datasets used and analysed during the current study are available from the corresponding author on reasonable request.

## Abbreviations

AMP: Acid mucopolysaccharides
BCVA: Best corrected visual acuity
OD: Right eye
OS: Left eye
PAS: Periodic acid–Schiff
PTK: Phototherapeutic keratectomy
